# Localization of brain networks activated by acupuncture at anatomically adjacent acupoints in healthy participants: neuroimaging evidence and implications for migraine and stroke

**DOI:** 10.3389/fnins.2025.1740153

**Published:** 2026-01-12

**Authors:** Zhiyang Zhang, Xu Ouyang, Chaorong Xie, Lichuan Zeng, Qixuan Fu, Qinyi Yan, Tong Wang, Xiao Wang, Ling Zhao

**Affiliations:** 1 School of Acupuncture and Tuina, Chengdu University of Traditional Chinese Medicine, Chengdu, Sichuan, China; 2 Department of Radiology, Hospital of Chengdu University of Traditional Chinese Medicine. Chengdu, Sichuan, China; 3 Key Laboratory of Acupuncture for Senile Disease (Chengdu University of TCM), Ministry of Education, Chengdu, China

**Keywords:** acupoint-specificity, functional connectivity network mapping, neurotransmitter, Yanglingquan, Zusanli

## Abstract

**Background:**

Neuroimaging investigations reveal heterogeneous acupuncture-induced brain activation patterns. Integrating acupoint-specific activation patterns into a unified connectomic framework enables systematic elucidation of acupoint-specific therapeutic mechanisms through network-level neural decoding.

**Methods:**

This study integrated functional connectivity network mapping (FCNM) methodology, canonical brain networks, and neurotransmitter distributions to delineate the distinct cerebral activation profiles of ST36 (Zusanli) and GB34 (Yanglingquan), two acupoints with anatomical proximity but divergent therapeutic indications in healthy controls (HCs).

**Results:**

The neural networks activated by acupuncture at ST36 and GB34 are both composed of widely distributed brain regions. These two acupoints co-activated the somatomotor network, the ventral attention network, and the dorsal attention network. The activation pattern of ST36 additionally emphasizes the visual network, while the activation pattern of GB34 primarily involves subcortical regions. The spatial patterns of activation brain networks of ST36 showed exploratory spatial correlations with the distributions of 6-fluoro-(18F)-L-3,4-dihydroxyphenylalanine (FDOPA), noradrenaline transporter (NET) and vesicular acetylcholine transporter (VAChT) neurotransmitter, while the GB34 were correlated with dopamine D1, dopamine D2, dopamine transporter, FDOPA, NET, *N*-methyl-D-aspartic acid receptor (NMDA), serotonin transporter (SERT), and VAChT neurotransmitter.

**Conclusion:**

This study delineates the distinct physiological mechanisms of ST36 and GB34 from neuroimaging and molecular perspectives. This discovery not only elucidates acupoint effect specificity through brain network organization but also expands our understanding of acupoint therapeutic mechanisms within the framework of systems neuroscience, providing a scientific basis for the precise application of acupuncture in treating diseases.

## Graphical Abstract

**Figure:**
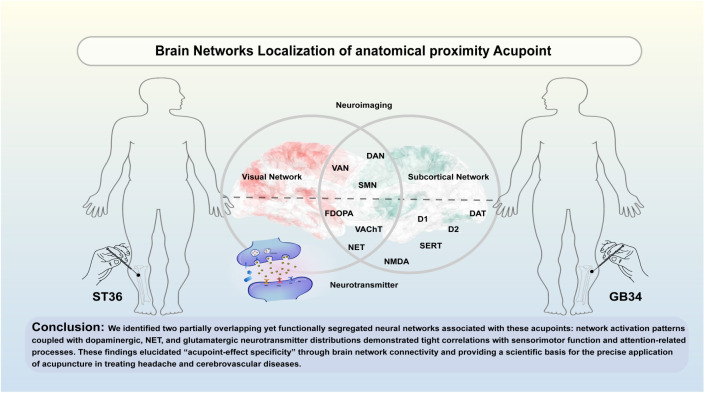
Localization of brain networks and spatial distribution of neurotransmitters in ST36 and GB34 following acupuncture in health participants.

## 1 Introduction

**FIGURE 1:**
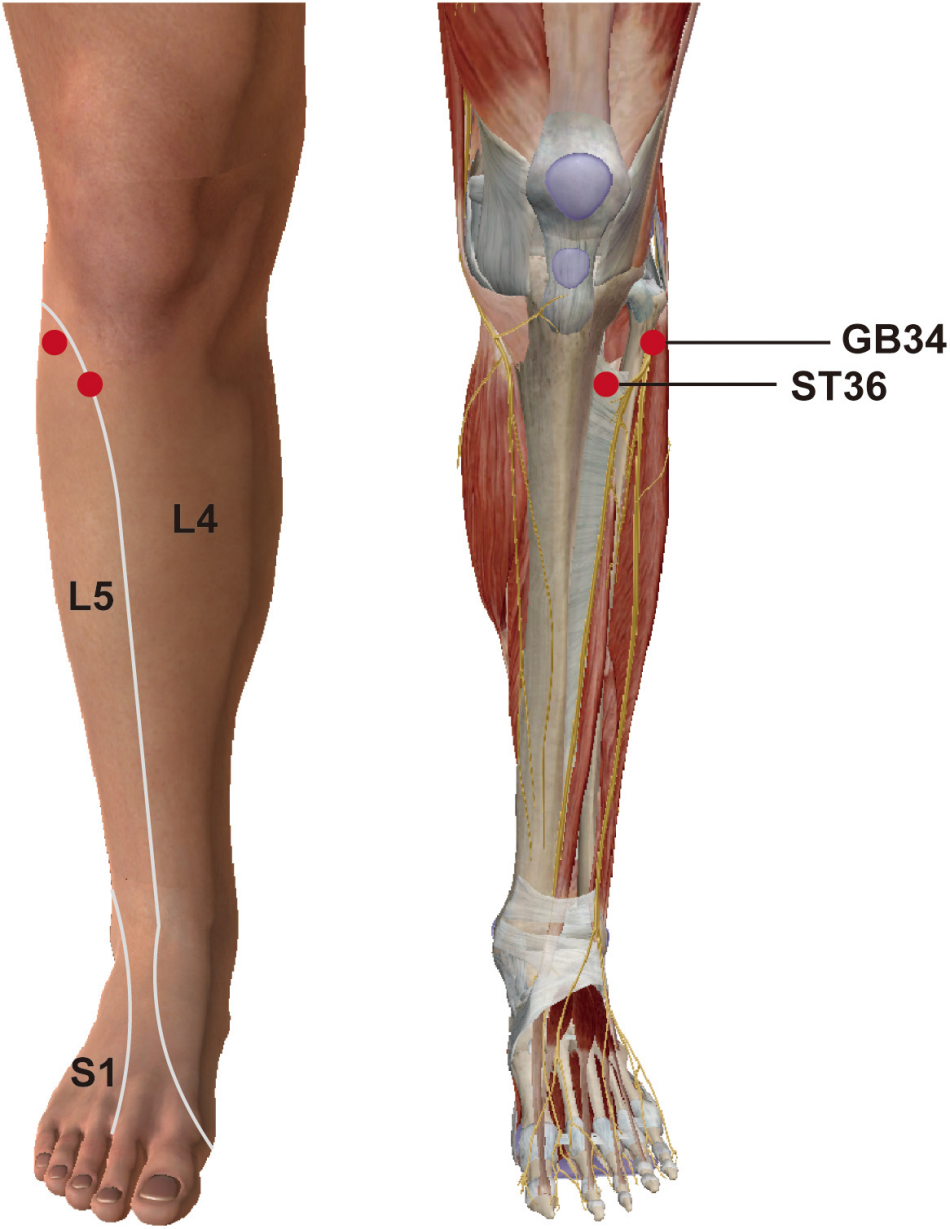
Location and neuro-segmental mapping of ST36 and GB34. ST36, Zusanli; GB34, Yanglingquan; L4, fourth lumbar vertebra; L5, fifth lumbar vertebra; S1, first sacral vertebra. Adapted from the “Human Anatomy Atlas 2018,” used under license purchased by Chengdu University of Traditional Chinese Medicine.

Acupuncture, an ancient technique in traditional Chinese medicine, has been widely accepted as a complementary therapy in modern medicine (Yu, 2020), yet the physiological mechanisms underlying different acupoints require further investigation. This study examines Zusanli (ST36) and Yanglingquan (GB34) to explore acupoint-specific brain networks. Although anatomically adjacent, their innervation differs: ST36 is innervated by the deep peroneal nerve (L4–L5), while GB34 is supplied by the common peroneal nerve (L5–S2) (Li S. H. et al., 2024; Yao et al., 2025) (Figure 1). Both acupoints alleviate lower limb dysfunction and pain (Chen et al., 2019; Tu et al., 2021), but their therapeutic profiles diverge. ST36 modulates gastrointestinal function and chronic fatigue, and is key in treating Yangming migraine (Allais et al., 2002; Cheng et al., 2017; Li et al., 2021; Pei et al., 2020), whereas GB34 focuses on post-stroke motor recovery and Shaoyang migraine (Jang et al., 2020; Jeun et al., 2005; Lu et al., 2023; Zhao et al., 2017). The specificity of these anatomically close yet therapeutically distinct acupoints remains unclear.

Advances in neuroimaging techniques have progressively elucidated the neural mechanisms underlying acupuncture’s multisystem integration, mediated through modulation of the somatomotor network (SMN), default mode network (DMN), limbic system, and cortico-basal ganglia circuitry (Kong et al., 2023; Wang et al., 2023; Zhou et al., 2023). Current neuroimaging evidence indicates that ST36 stimulation activates the anterior cingulate, insular, brainstem and enhances DMN connectivity (Wang et al., 2024; Zhou et al., 2023), while GB34 modulates the motor cortex, cerebellum, and basal ganglia (Lee et al., 2024; Ning et al., 2017). However, small sample sizes and methodological inconsistencies have led to heterogeneous findings and insufficient evidence for “acupoint specificity” (Huang et al., 2022), particularly in the context of their distinct clinical efficacy for conditions like migraine and stroke.

To address these gaps, the functional connectivity network mapping (FCNM) method integrates spatially heterogeneous brain regions with large-scale connectome data to establish robust brain–behavior correspondences. Recent studies employing this methodology have successfully delineated aberrant neural networks associated with symptom dimensions and illness stages of schizophrenia, major depressive disorder, and suicidality-related pathologies (Cheng et al., 2025; Li J. et al., 2024; Xu et al., 2024; Zhang et al., 2024). Therefore, the novel methodological framework simultaneously elucidating the distinct neuroimaging signatures of ST36 and GB34 acupoints has the potential to reveal the acupoint-specificity from the perspective of neuroimage. Previous studies have demonstrated that acupuncture can regulate cerebral neurotransmitter levels, such as alleviating pain through modulation of GABA and opioid receptors (Harris et al., 2009; Mawla et al., 2021), reducing drug addiction by modulating dopamine pathways (Lee et al., 2021). Dysregulation of these neurotransmitters is also a hallmark of both chronic headache pathologies and the stroke (Alves et al., 2025; Salinas-Abarca et al., 2025). The JuSpace toolbox^1^ provides a cross-modal assessment framework for neuroimaging and neurotransmitter mapping (Dukart et al., 2021). Therefore, this study proposes to investigate the physiological mechanisms underlying acupoint specificity from both neuroimaging and neurochemical perspectives.

In summary, this study integrates FCNM with systematic review to characterize neuroimaging signatures of ST36 and GB34 in healthy individuals. Combining canonical brain network and neurotransmitter analyses, we hypothesize that these anatomically adjacent but meridian-distinct acupoints exhibit shared and distinct network responses associated with their efficacy. A clear delineation of these networks is anticipated to provide a mechanistic explanation for their traditional use in managing headache and promoting recovery after cerebrovascular events, ultimately contributing to a more precise understanding of acupoint specificity.

## 2 Materials and methods

### 2.1 Registration

This study was registered on the PROSPERO^2^, with the registration number of CRD420251082684.

### 2.2 Search strategy

A systematic literature retrieval was conducted in the database of PubMed, Web of Science, Excerpta Medica Database (EMBASE), The Cochrane Library, China National Knowledge Infrastructure (CNKI), Wan fang Database, and Chongqing VIP, Chinese Biomedical Literature Database (CBM) from inception to March 2025, according to Preferred Reporting Items for Systematic reviews and Meta-Analysis (PRISMA) (Page et al., 2021). The following four sets of search terms were adopted in English: (“acupuncture” or “acupuncture therapy” or “electroacupuncture” or “EA”) and (“zusanli” or “ST36” or “yanglinquan” or “GB34”) and “magnetic resonance imaging” or “neuro-imaging” or “fMRI” or “BOLD” or “FC” or “ALFF” or “ReHo” or “functional connectivity” or “amplitude Low-frequency fluctuation” or “regional homogeneity”). Relevant Chinese search terms were also searched. Then, we will manually screen the literature of the retrieved studies and reviews.

### 2.3 Inclusion and exclusion criteria

In this review, we included all studies that used fMRI to investigate the effect of acupuncture on the human brain. Studies meeting the following criteria were eligible for inclusion in this systematic review:

(1)    They had to include HCs only;
(2)    They needed to report findings related to ST36, GB34.
(3)    They had to report findings in 3D coordinates in either the Montreal Neurological Institute (MNI) space (Evans et al., 1992) or Talairach space (Talairach and Tournoux, 1988);
(4)    They had to incorporate whole-brain signaling analysis.

Studies were excluded if they:

(1)    Did not include a HCs group;
(2)    Investigated only region of interest (ROI) results or used analytical methods such as functional connectivity, independent component analysis, or graph theory;
(3)    Consisted of reviews, case reports, conference abstracts, or animal studies;
(4)    Did not report peak effect coordinates;
(5)    Were not available in full text;
(6)    Had a sample size of fewer than 10 participants were excluded in FCNM analysis.

### 2.4 Data extraction

The two authors (ZZ and OY) independently extracted data from the literature that met the inclusion criteria. The extracted contents included basic information of the literature (publication year, author, sample size, nation), basic characteristics of the study subjects (age, gender), and technical details of MRI (MRI scanner, method analysis, imaging results). The above information is shown in Supplementary Tables 1, 2. Any discrepancies were discussed with the third researcher until a consensus was reached.

## 3 Data analysis

### 3.1 Discovery and replication datasets

Our work employed Southwest University Longitudinal Imaging Multimodal (SLIM) as a discovery dataset^3^ and 1,000 Functional Connectomes Project (FCP) of Cambridge Buckner database^4^ as a cross-scanner replication dataset.

### 3.2 fMRI data acquisition and preprocessing

Resting-state functional magnetic resonance imaging (fMRI) data were acquired for SLIM and FCP participants using 3T Siemens Trio scanners. Preprocessing of the fMRI data was performed with DPABI version 4.3^5^, following a standardized pipeline that included the following steps: (1) removal of the initial five time points to minimize the impact of scanner instability and facilitate participant adaptation to the scanning environment; (2) slice timing correction to account for inter-slice acquisition time differences; (3) realignment for head motion correction; (4) spatial normalization to Montreal Neurological Institute (MNI) space via deformation fields derived from structural image segmentation, with resampled voxel dimensions of 3 mm^3^; (5) spatial smoothing using a Gaussian kernel with a 6 mm full-width at half maximum; (6) linear detrending of the time series; (7) regression of nuisance covariates, including Friston-24 motion parameters, white matter signal, cerebrospinal fluid signal, and global signal; (8) band-pass filtering within the 0.01–0.08 Hz frequency range. Participants exhibiting excessive head motion—defined as translational displacement greater than 2.0 mm or rotational motion exceeding 2.0° were excluded from the analysis. Additional exclusions were applied in cases of incomplete brain coverage or suboptimal image quality. Detailed scanning parameters are provided in Supplementary Table 3.

### 3.3 Functional connectivity network mapping

Based on activation coordinates derived from healthy volunteers that reflect inter-individual differences, the FCNM approach was applied to construct central response network models for the ST36 and GB34 acupoints. The procedure involved the following steps: First, spherical regions with a 4-mm radius were created around each coordinate corresponding to a given contrast, and these were merged to form a combined seed mask specific to that contrast (referred to hereafter as the contrast seed). Second, using preprocessed resting-state fMRI data from the SLIM dataset, participant-level contrast seed-to-whole-brain functional connectivity (FC) maps were generated by computing Pearson’s correlation coefficients between the time series of the contrast seed and those of every voxel across the whole brain. The resulting correlation values were then subjected to Fisher’s z-transformation to approximate a normal distribution. Third, the 573 individual-level FC maps were submitted to a voxel-wise one-sample *t*-test to identify brain regions exhibiting significant functional connectivity with each contrast seed. It should be noted that only positive functional connectivity was considered, as the biological interpretation of negative correlations remains contentious. Fourth, the resulting group-level t-maps were thresholded at a significance level of *P* < 0.01, corrected for multiple comparisons using a voxel-wise false discovery rate (FDR) procedure, and subsequently binarized. Finally, the binarized maps were overlaid to create composite network probability maps. These were thresholded at a 60% probability level to produce the final central response network models for the ST36 and GB34 acupoints.

### 3.4 Relationship to canonical brain networks

For ease of interpretability, we investigated the spatial relations between one brain abnormality networks of healthy volunteers and 8 well-established canonical brain networks. The seven cortical networks were defined as the visual, somatomotor, dorsal attention, ventral attention, limbic, frontoparietal control, and default networks according to the Yeo et al. (2011) study. The Brainnetome atlas (Fan et al., 2016) was adopted to defined the subcortical regions including the thalamus, caudate, putamen, pallidum, hippocampus, amygdala and nucleus accumbens. The proportion of overlapping voxels between each brain abnormality network and a canonical network to all voxels within the corresponding brain abnormality network was calculated to assess their spatial relation.

### 3.5 Spatial correlation with neurotransmitter density maps

The JuSpace toolbox (version 2.0, see text footnote 1) covering various neurotransmitter maps helped assess associations of spatial patterns of functional activation in HCs with specific neurotransmitter systems. The following neurotransmitter systems were considered: (Yu, 2020) serotonin transmission: serotonin 5-hydroxytryptamine receptor subtype 1a (5HT1a), serotonin 5-hydroxytryptamine receptor subtype 1b (5HT1b), serotonin 5-hydroxytryptamine receptor subtype 2a (5HT2a), and serotonin transporter (SERT); (2) dopamine transmission: dopamine D1 (D1), dopamine D2 (D2), dopamine transporter (DAT), and 6-fluoro-(18F)-L-3,4-dihydroxyphenylalanine (FDOPA); (3) gamma-aminobutyric acid type a (GABAa); (4) Opioid receptor: kappa opioid receptor (KappaOp), μ-opioid receptor (MU), (5) noradrenaline transporter (NAT); (6) cannabinoid 1 receptor (CB1); (7) N-methyl-D-aspartic acid receptor (NMDA); (8) vesicular acetylcholine transporter (VAChT), (9) metabotropic glutamate receptor 5 (mGluR5). The neurotransmitter/metabolic density maps selected were the most commonly used in the literature and were derived from the largest number of HCs.

Based on the FCNM maps of ST36 and GB34, the JuSpace toolbox (computing option 3) and the default Neuromorphometrics Atlas comprising 119 regions were employed to assess spatial correspondence with selected neurotransmitter receptor/transporter maps. Specifically, Fisher’z-transformed Spearman correlation coefficients were computed between each FCNM map and the spatial distributions of the respective receptors or transporters. To account for spatial autocorrelation, a gray matter probability map was incorporated as a covariate. Statistical significance of the correlation coefficients was evaluated using one-sample t-tests against zero, with exact *P*-values derived from 10,000 permutations. Multiple comparisons were controlled by applying FDR correction at a threshold of *P* < 0.05.

### 3.6 Validation analyses

To evaluate the robustness of our findings, several validation analyses were performed. First, the FCNM procedure was repeated using spherical seeds with radii of 1 and 7 mm to examine the influence of seed size. Second, the same analytical process was applied to an independent replication dataset (i.e., the FCP) to assess the impact of dataset selection. To quantitatively evaluate the spatial similarity between FCNM maps, the Dice coefficient—defined as 2 × (number of overlapping voxels)/(total voxels in network 1 + total voxels in network 2)—was computed, with higher values indicating greater spatial resemblance. In line with the established consensus that Dice coefficients between 0.5 and 1 reflect meaningful spatial overlap, a one-sample *t*-test was conducted to determine whether the obtained Dice coefficients were significantly lower than 0.5.

### 3.7 Acupoint specificity testing

To test the acupoint specificity of central response network, we selected ST36 and GB34 as contrasting acupoints based on their anatomical proximity yet distinct meridian affiliations.

## 4 Result

### 4.1 Literature selection process

**FIGURE 2:**
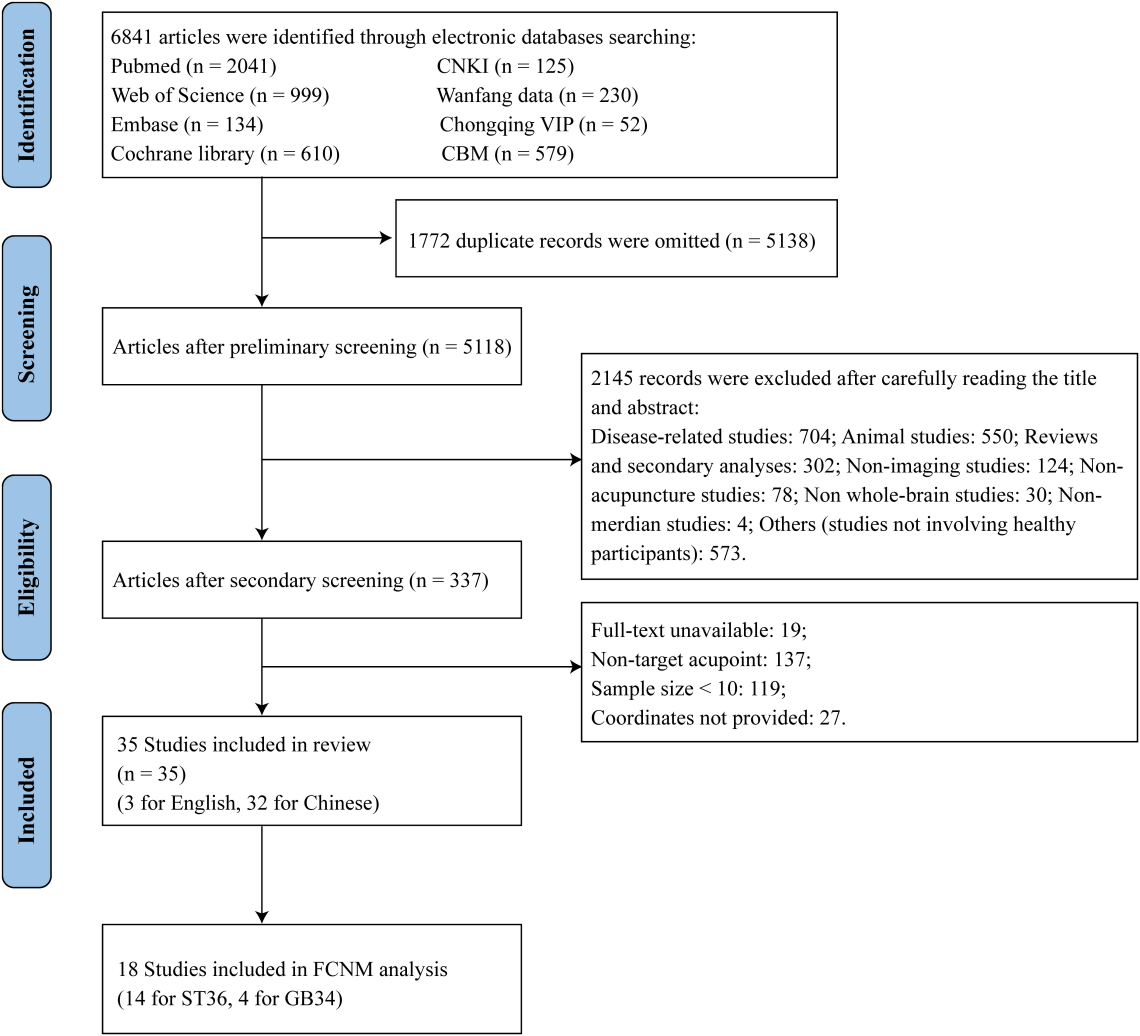
Flow chart describing the study selection process. CNKI, China National Knowledge Infrastructure; CBM, Chinese Biomedical Literature Database; VIP, the China Science and Technology Journal Database.

A total of 6,841 studies were retrieved from the database for review. Initially, 1,722 duplicate studies were excluded. Subsequently, 4,781 studies were excluded after a preliminary screening of the remaining 5,118 studies [2,415 excluded after reading abstracts, 704 disease-related studies, 550 animal studies, 302 reviews and secondary analyses, 124 non-imaging studies, 78 non-acupuncture studies, 30 non-whole-brain studies, 4 non-meridian studies, and 573 others (studies not involving healthy participants)]. Following this, 302 studies were excluded after a full-text review (137 studies involving non-target acupoints, 119 studies with a study population of fewer than 10 participants, and 27 studies with unavailable coordinates). Ultimately, 35 studies (Bao et al., 2010; Bi et al., 2017; Cai et al., 2007; Chen and Liu, 2019; Chen and Liu, 2019; Cheng and Wang, 2014; Dong et al., 2013; Duan et al., 2012; Fang et al., 2006, 2012; Long et al., 2016; Lu et al., 2017; Na et al., 2009; Shang and Li, 2017; Tan et al., 2009, 2013; Tian et al., 2009; Wu et al., 2016; Xiao et al., 2008; Yeo et al., 2016; Yuan and Huang, 2015; Zhang et al., 2016; Zhang, 2011; Zhu et al., 2016) were included in this review, with 18 studies incorporated into FCNM analysis (including 14 focusing on ST36 and 4 on GB34), as illustrated in Figure 2, the PRISMA flow chart.

### 4.2 Study characteristics

This study included a total of 35 articles, all of which were incorporated into the systematic review and imaging analysis. Among these, 29 studies focused on imaging research related to acupuncture at the ST36 acupoint, while five studies investigated the GB34 acupoint, one study both investigated ST36 and GB34 acupoints. The total number of HCs included was 513 for ST36 and 112 for GB34. The age range of the HCs spanned from 19 to 36 years. The majority of the studies were conducted in China (33 studies), with one study each from South Korea and the United States. The primary acupuncture modalities employed were manual acupuncture (28 studies), electroacupuncture (6 studies), and moxibustion (1 study). Additionally, among the included studies, 20 utilized 1.5T MRI scanning, 13 utilized 3.0T MRI scanning, and two studies did not report the scanning method used. Furthermore, in the 14 included neuroimaging studies focusing on ST36, the top ten most frequently involved brain regions were ranked as follows: anterior cingulate cortex, thalamus, hippocampus, temporal lobe, postcentral gyrus, insula, middle frontal gyrus, parahippocampal gyrus, and amygdala. Among the 4 included studies investigating GB34, the most prominently engaged brain regions were identified in the frontal lobe, putamen, caudate and thalamus.

### 4.3 Activation network of ST36

**FIGURE 3:**
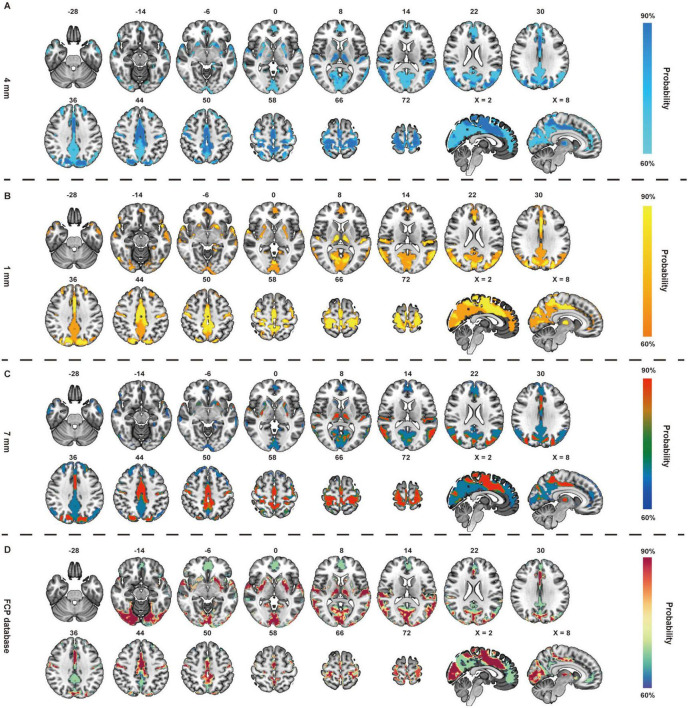
Brain activated network of ST36 in healthy controls (HCs). The radius spheres of seed size with 4 mm **(A)**, 1 mm **(B)**, 7 mm **(C)**, and another dataset of Functional Connectomes Project (FCP) **(D)** are shown as the network probability maps thresholded at 60%, indicating brain regions functionally connected to more than 60% of the contrast seeds.

The activated network of ST36 comprised a broadly distributed set of brain areas principally encompassing the precentral gyrus, postcentral gyrus, precuneus, cingulate gyrus, prefrontal lobe, temporal lobe, occipital lobe and thalamus (Figure 3). In terms of canonical brain networks, the activated network predominantly implicated the somatomotor network (SMN) (overlapping proportion: 38.45%), ventral attention network (VAN) (15.18%), visual (14.37%), dorsal attention network (DAN) (13.98%), default mode network (DMN) (6.97%), subcortical regions (6.77%), limbic (2.85%), frontoparietal control (1.44%) networks (Figure 5A). The ST36 activation networks were significantly correlated with exploratory spatial distribution of FDOPA (rho = 0.4425, *P* < 0.001), NAT (rho = 0.6226, *P* < 0.001), VAChT (rho = 0.2815, *P* = 0.0139) (Table 1 and Figure 5B).

**TABLE 1:** Spatial correlation analyses between ST36 and GB34 brain activation and ne-urotransmitter/metabolism distribution.

Neurotransmitter activity maps	Fisher’s Z Spearman rho	*P*-value (FDR corrected)
**ST36**		
5HT1a	0.0282	0.8296
5HT1b	−0.2071	0.0765
5HT2a	−0.1678	0.3446
CB1	0.1632	0.3509
D1	0.0890	0.4176
D2	0.1676	0.1308
DAT	0.2264	0.0544
FDOPA	0.4425	**0.0008**
GABAa	0.0417	0.8296
KappaOp	0.2629	0.1547
MU	−0.0885	0.8296
NET	0.6226	**0.0008**
NMDA	0.2907	0.0765
SERT	0.1817	0.1207
VAChT	0.2815	**0.0139**
mGluR5	0.2621	0.1308
**GB34**		
5HT1a	−0.1702	0.2202
5HT1b	0.1217	0.2202
5HT2a	−0.1676	0.2338
CB1	0.2231	0.1475
D1	0.2982	**0.0026**
D2	0.2244	**0.0280**
DAT	0.2963	**0.0037**
FDOPA	0.5792	**0.0008**
GABAa	0.0486	0.7501
KappaOp	0.3178	0.0645
MU	0.3713	0.1309
NAT	0.3698	**0.0008**
NMDA	0.4587	**0.0008**
SERT	0.2566	**0.0119**
VAChT	0.4208	**0.0008**
mGluR5	0.2618	0.0938

ST36, Zusanli; GB34, Yanglingquan; 5HT1a, serotonin 5-hydroxytryptamine receptor subtype 1a; 5HT1b, serotonin 5-hydroxytryptamine receptor subtype 1b; 5HT2a, serotonin 5-hydroxytryptamine receptor subtype 2a; SERT, serotonin transporter; D1, dopamine D1; D2, dopamine D2; DAT, dopamine transporter; FDOPA, 6-fluoro-(18F)-L-3,4-dihydroxyphenylalanine; GABAa, gamma-aminobutyric acid type a; KappaOp, Opioid receptor, kappa opioid receptor; MU, μ-opioid receptor; NAT, noradrenaline transporter; CB1, cannabinoid 1 receptor; NMDA, *N*-methyl-D-aspartic acid receptor; VAChT, vesicular acetylcholine transporter; mGluR5, metabotropic glutamate receptor 5. The bold values indicated results corrected for false discovery rate *P* < 0.05.

### 4.4 Activation network of GB34

**FIGURE 4:**
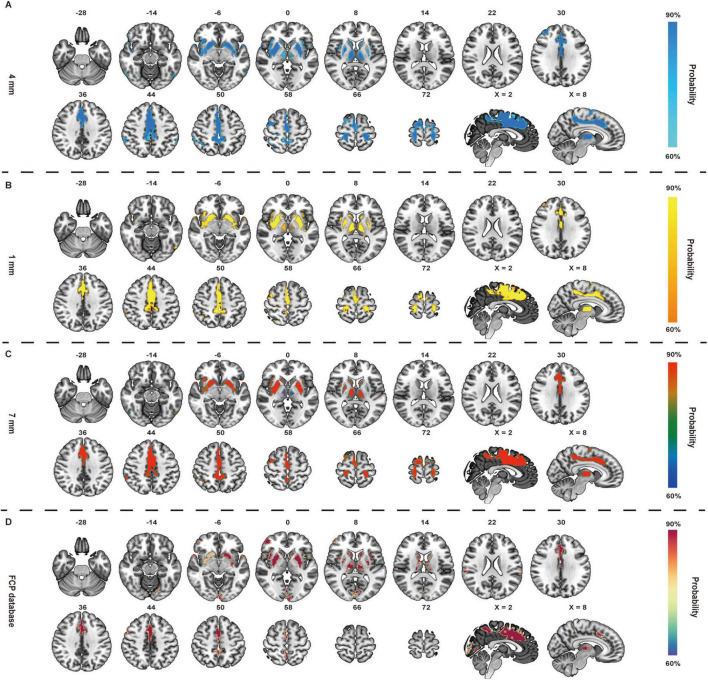
Brain activated network of GB34 in healthy controls (HCs). The radius spheres of seed size with 4 mm **(A)**, 1 mm **(B)**, 7 mm **(C)**, and another dataset of Functional Connectomes Project (FCP) **(D)** are shown as the network probability maps thresholded at 60%, indicating brain regions functionally connected to more than 60% of the contrast seeds.

**FIGURE 5:**
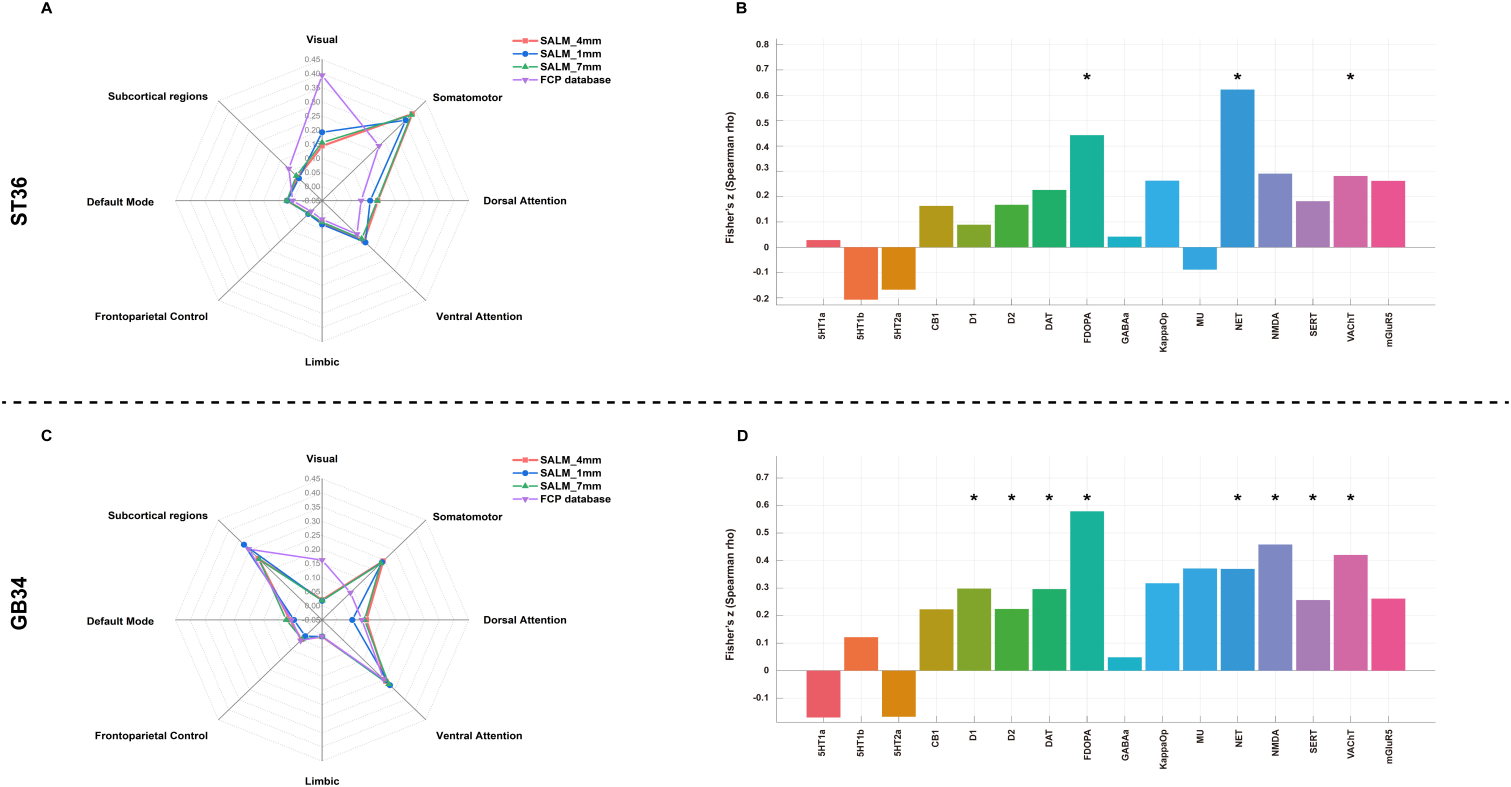
Brain activated networks of ST36 and GB34 in relation to canonical brain networks and Neurotransmitter system. **(A)** ST36 network. **(B)** The spatial correlation between ST36 network and neurotransmitter density maps. **(C)** GB34 network. **(D)** The spatial correlation between ST36 network and neurotransmitter density maps. Polar plots show the proportion of overlapping voxels between each brain abnormality network and a canonical network to all voxels within the corresponding brain abnormality network. *Indicate results corrected for false discovery rate (FDR) *P* < 0.05.

The activated network of GB34 comprised a broadly distributed set of brain areas principally encompassing the precentral gyrus, postcentral gyrus, prefrontal lobe, putamen, insula, caudate nucleus, thalamus, cingulate gyrus, supplementary motor area (Figure 4). In terms of canonical brain networks, the activated network predominantly implicated VAN (overlapping proportion: 25.78%), subcortical regions (25.75%), the SMN (24.45%), DAN (10.11%), DMN (5.99%), frontoparietal control (4.91%), visual (2.09%), and limbic (0.92%) networks (Figure 5C). The GB34 activation networks were significantly correlated with exploratory spatial distribution of D1 (rho = 0.2982, *P* = 0.0026), D2 (rho = 0.2244, *P* = 0.0280), DAT (rho = 0.2963, *P* = 0.0037), FDOPA (rho = 0.5792, *P* < 0.001), NAT (rho = 0.3698, *P* < 0.001), NMDA (rho = 0.4587, *P* < 0.001), SERT (rho = 0.2566, *P* = 0.0119), VAChT (rho = 0.4208, *P* < 0.001) (Figure 5D and Table 1).

### 4.5 Validation analyses

First, when repeating the FCNM procedure with 1- and 7-mm radius spheres, we observed that the resulting brain networks of ST36 were nearly identical to those with the 4-mm-radius sphere (4- vs. 1-mm Dice coefficients: 0.83; 4- vs. 7-mm Dice coefficients: 0.90); and the result brain network of GB34 (4- vs. 1-mm Dice coefficients: 0.88; 4- vs. 7-mm Dice coefficients: 0.94). Second, we observed that the brain activation networks of ST36 and GB34 derived from the replication dataset (FCP) were consistent with those from the discovery dataset (SLIM), with minor differences possibly arising from variation in sample sizes (Dice coefficients: 0.56, 0.51). Furthermore, the sensitivity analysis of task-fMRI based study demonstrated high spatial consistency (Supplementary Figure 1).

## 5 Discussion

By integrating the novel FCNM method with large-scale human brain connectome data, this study systematically investigated the brain network localization and neurotransmitter distribution of ST36 and GB34 (two acupoints with similar anatomical positions but distinct therapeutic effects). The results demonstrated that although both acupoints activate widely distributed brain regions, including the VAN, DAN, and SMN, ST36 exhibits stronger modulation of the visual network, whereas GB34 primarily involves subcortical regions. Additionally, the spatial patterns of activated brain network of ST36 and GB34 were correlated with the spatial distribution of dopamine transmission, noradrenaline transporter, and acetylcholinergic neurotransmitters, the GB34 was also correlated with the spatial distribution of serotonin and glutamate receptors neurotransmitters.

### 5.1 Emerging paradigms in acupuncture neuroimaging research

With the advancement of neuroimaging technologies, fMRI has been widely used to explore the central response mechanisms of acupuncture. Previous studies have highlighted that factors such as sample size and research methodologies contribute to high heterogeneity in findings, raising concerns about scientific validity and reproducibility (Claunch et al., 2012; Zhang et al., 2023). Given the multidimensional regulatory characteristics of acupoint effects, recent research increasingly emphasizes moving beyond single brain regions to focus on interactions within brain networks (Cai et al., 2018; Jiang et al., 2024; Liu et al., 2022). The development of large-scale human brain connectome data and neuroimaging methods like FCNM (which maps lesions from diverse brain locations to a common neural network) offers potential solutions to address heterogeneity and low reproducibility. Since acupuncture effects are closely tied to central network dynamics, we applied this methodology to investigate the activated brain networks of ST36 (the most studied acupoint in current literature) and GB34 (an anatomically adjacent but meridian-distinct acupoint). The findings were further interpreted through alignment with canonical brain networks and neurotransmitter distribution.

### 5.2 Co-activated brain networks of ST36 and GB34 acupuncture points

As an invasive somatosensory stimulus, acupuncture has been documented to modulate sensorimotor networks (Cai et al., 2018; Claunch et al., 2012), a recent study based on neural tracing technology exploring the neural pathways of acupuncture has shown that acupoints exhibit common neural projections to regions such as the primary motor cortex and the secondary motor cortex (Liang et al., 2025), our FCNM-based functional mapping analysis corroborates these findings, demonstrating extensive SMN activation during stimulation of both ST36 and GB34. Clinical studies demonstrate that acupuncture can alleviate pain and improve functional impairments by modulating functional activities within the sensorimotor network (Chen et al., 2023; Qian et al., 2025). This shared SMN modulation provides a common neural substrate for their efficacy in both pain conditions like headache and motor rehabilitation post-stroke. However, the therapeutic emphasis diverges: the SMN activation by ST36 may be more linked to modulating the sensory-discriminative aspects of headache pain, whereas for GB34, SMN integration is paramount for restoring motor function, a core deficit in cerebrovascular diseases. These findings may partially elucidate the physiological mechanisms underlying the therapeutic effects of acupoint stimulation. However, whether similar co-activation patterns in sensorimotor networks exist with other acupoints requires further investigation through systematic comparative studies employing standardized neuroimaging paradigm.

Furthermore, our investigation identified the activation of the VAN and the DAN within the brain’s neural circuitry in response to stimulation of the ST36 and GB34 acupuncture points. The VAN is primarily associated with bottom-up attention allocation, while the DAN is involved in goal-directed, top-down attention regulation (Maillet et al., 2019). Additionally, the neurotransmitter primarily implicated in these two attention networks is norepinephrine (NET) (Shine, 2019). Our findings also suggest that the brain network activation associated with ST36 and GB34 correlates with a high distribution of NET. Previous studies have demonstrated that NET expression is downregulated in individuals with attention deficit disorder (Thiele and Bellgrove, 2018; Wiesman et al., 2025), establishing a link between NET and attention. Notably, the noradrenergic system is not only crucial for attention but also plays a significant role in post-stroke recovery and the pathophysiology of certain headaches (Wang et al., 2020; Zahrai et al., 2020). Consequently, the co-modulation of the attention networks and the noradrenergic system by both acupoints represents a convergent pathway for treating distinct disorders, primarily targeting headache pathology through sensory-attentional integration, while simultaneously supporting cognitive-attentional components of recovery in cerebrovascular patients.

### 5.3 ST36-specific modulation brain network

Our study demonstrated that acupuncture at ST36 elicits activation in the visual network. However, traditional Chinese medicine (TCM) does not describe ST36 as having therapeutic effects related to vision. While previous literature on the central responses to ST36 stimulation has occasionally reported activations in occipital lobe-related regions (Hu et al., 2025), no studies have specifically investigated the relationship between ST36 and the visual network. The functional connectivity map generated in our study primarily involved the visual network, whereas GB34 did not exhibit such an association. This finding suggests that ST36 may have a specific regulatory effect on the visual network, potentially expanding our understanding of its therapeutic mechanisms—though further clinical studies are needed for validation. Moreover, recent evidence indicates that the visual network plays a role in attentional modulation and allocation, forming part of the brain’s attention system alongside the VAN and DAN. Studies have shown that the attention system is functionally linked to autonomic nervous system (ANS) activity (Barber et al., 2020; Lin et al., 2020), chronic digestive disorders, such as irritable bowel syndrome and inflammatory bowel disease, are associated with altered ANS function (Rubio et al., 2015), high-quality randomized controlled trials (RCTs) have demonstrated that ST36, as a primary acupoint, can modulate gastrointestinal activity (Pei et al., 2025; Zhao et al., 2024). Therefore, we hypothesize that acupuncture at ST36 may regulate autonomic function by modulating the attentional network, thereby improving gastrointestinal symptoms. Furthermore, this ST36-specific visual-attention network integration provides a unique mechanistic explanation for its therapeutic profile. It strongly supports its use in treating headache disorders, particularly migraine with visual aura or gastrointestinal comorbidities (e.g., nausea) by modulating the neural circuits that process these specific symptoms. In contrast, its role in cerebrovascular diseases is more supportive and indirect, potentially aiding in the recovery of overall autonomic balance and well-being, rather than targeting core motor deficits.

### 5.4 GB34-specific modulation brain network

GB34 is primarily used to treat movement disorder-related diseases and pain such as post-stroke limb motor dysfunction, Parkinson’s disease and migraine (Chen et al., 2015; Zeng and Zhao, 2016; Zhao et al., 2017). Our findings demonstrated that GB34 specifically activates subcortical brain networks involving the basal ganglia, which are closely associated with movement disorders and pain signal transmission (Li et al., 2022; Motl et al., 2021). This specific subcortical targeting starkly differentiates its primary clinical role from ST36, positioning GB34 as a key acupoint for addressing the core motor impairments resulting from cerebrovascular diseases. Moreover, the activation of subcortical brain networks was found to correlate with high distributions of dopaminergic, NMDA, and SERT-related neurotransmitters. Parkinson’s disease and other movement disorders are linked to dysregulation of dopaminergic neurotransmission (Cramb et al., 2023), while NMDA, as an excitatory neurotransmitter, serves as a biomarker of neuronal integrity (Foerster et al., 2013), SERT agonists have been shown to alleviate involuntary movements in Parkinson’s patients (Pagano et al., 2017). Crucially, the serotonin (5-HT) system, implicated by the SERT correlation, is profoundly involved in the pathophysiology of headache disorders, particularly migraine (de Vries et al., 2020). Thus, we elucidate that GB34 possesses a unique therapeutic spectrum, primarily targeting motor deficits in cerebrovascular diseases with efficacy on migraine, achieved through the integrated modulation of subcortical networks and a broader neurochemical profile.

While our findings identify distinct brain network targets for ST36 and GB34 in healthy individuals, translating these into clinical practice and guide the optimization of stimulation parameter requires further exploration. Future research should validate whether modulating these specific networks correlates with symptom improvement in patients.

Current study has several limitations that warrant acknowledgment. Firstly, while resting-state data provide a stable framework for mapping inter-individual network architectures, they do not capture the immediate, dynamic neural responses elicited during acupuncture stimulation, future studies should incorporate task-fMRI paradigms to delineate the neural dynamics of acupuncture. Secondly, due to the lack of neuroimaging data, we were unable to perform validation analyses on acupuncture stimulation at ST36 and ST34. Future studies will incorporate post-acupuncture imaging data to assess the stability of our findings. Secondly, as JuSpace utilizes nuclear imaging-derived neurotransmitter profiles from healthy individuals and the results of the JuSpace can only indicate the spatial similarity between activation networks and neurotransmitter distribution, and cannot be directly reflected actual changes in neurotransmitter levels. Thirdly, our study was conducted in healthy participants and did not directly explore the pathological mechanisms of specific diseases such as migraine and stroke. Therefore, whether the acupoint-specific networks we identified are consistent with the impaired networks in the diseases they treat, and how they exert therapeutic effects by rectifying these specific pathological changes, remains to be validated in future clinical studies involving patient populations.

## 6 Conclusion

In conclusion, this study integrates the FCNM approach with large-scale human connectome data and neurotransmitter mapping to delineate the distinct physiological mechanisms underlying ST36 and GB34, acupoints with anatomical proximity but divergent therapeutic profiles from neuroimaging and molecular perspectives. This discovery not only elucidates acupoint effect specificity through brain network organization but also provides a scientific framework for precision acupoint selection in treating diseases, based on their shared and distinct central mechanisms.

## Data Availability

The original contributions presented in this study are included in this article/Supplementary material, further inquiries can be directed to the corresponding authors.

## Footnotes

1
https://github.com/juryxy/JuSpace

2
https://www.crd.york.ac.uk/PROSPERO/

3
https://fcon_1000.projects.nitrc.org/indi/retro/southwestuni_qiu_index.html

4
https://fcon_1000.projects.nitrc.org/fcpClassic/FcpTable.html

5
http://rfmri.org/dpabi
