# Supplementary material for: Localization of brain networks activated by acupuncture at anatomically adjacent acupoints in healthy participants: neuroimaging evidence and implications for migraine and stroke
Source: Front Neurosci. 2026 Jan 12;19:1740153. doi: 10.3389/fnins.2025.1740153 (PMC12833230; doi:10.3389/fnins.2025.1740153)
Supplement: Supplementary file 1 [file Data_Sheet_1.pdf]

Table S1. Sample and imaging characteristics of the studies included in the resting-state activity analysis of ST36.

Study	Male/Female	Age (years)	Intervention modalities		Scanner	Measure
			Intervention	Operational details		
Bao et al.2010	6 / 9	23.3	Acupuncture	Even reinforcing and reducing manipulation the needle insertion depth is 2.5 cm.	1.5T MRI	Bold signal
Fen et al.2017	9 / 11	26.9 ± 2.6	Moxibustion	Different moxibustion distances of 2 cm, 3 cm, 4 cm, and 5 cm.	3.0T MRI	fALFF、ReHo
Dong et al.2013	8 / 8	19 – 25	Acupuncture	Twirling Reinforcing and Reducing, the needle insertion depth is 1.5 – 2.5 cm.	3.0T MRI	Bold signal

Duan et al.2012	8 / 8	25 ± 3.3	Acupuncture	Twirling Reinforcing and Reducing, the needle insertion depth is 1.5 cm.	1.5T MRI	ReHo
Jiang et al.2010	7 / 6	23.75 ± 4.3	Electroacupuncture	5 Hz, the needle insertion depth is 3 cm.	1.5T MRI	Bold signal
Li et al.2013	7 / 8	23.6 ± 2.2	Acupuncture	Even reinforcing and reducing manipulation the needle insertion depth is 2.5 cm.	3.0T MRI	ALFF
Li et al.2013	7 / 8	23.6 ± 2.2	Acupuncture	Even reinforcing and reducing manipulation the needle insertion depth is 2.5 cm.	3.0T MRI	ReHo
Long et al.2008	7 / 10	24.6 ± 0.3	Acupuncture	Twirling Reinforcing and Reducing	1.5T MRI	ALFF

Wu et al.2011	8 / 8	25 ± 3.3	Acupuncture	Lifting-thrusting reinforcing and reducing, the needle insertion depth is 1.5 cm.	MRI	ReHo
Zhu et al.2016	10 / 10	25.55 ± 2.16	Acupuncture	Twirling Reinforcing and Reducing, the needle insertion depth is 2.5 cm.	3.0T MRI	ASL
Fu et al.2013	5 / 5	23 – 25	Acupuncture	Twirling Reinforcing and Reducing	1.5T MRI	Bold signal
Liu et al.2014	4 / 6	20 – 34	Acupuncture	Twirling Reinforcing and Reducing	3.0T MRI	ReHo
Kathleen et al.2005	8 / 7	29.8 ± 7.5	Acupuncture	Twirling Reinforcing and Reducing, the needle insertion depth is 2 - 3 cm.	1.5T MRI	Bold signal
Lu et al.2017	17 / 0	24.0 ± 1.2	Acupuncture	Lifting-thrusting reinforcing and reducing, the needle insertion depth is 3 cm.	3.0T MRI	Bold signal

Cai et al.2007	6 / 1	35.86	Acupuncture	Twirling Reinforcing and Reducing	1.5T MRI	Bold signal
Chen et al.2019	9 / 9	21.8 ± 1.6	Acupuncture	Manipulation, the needle insertion depth is 1.5 cm.	1.5T MRI	Bold signal
Chen et al.2010	9 / 6	24.5 ± 0.6	Acupuncture	The needle insertion depth is 4 cm.	1.5T MRI	ALFF
Cheng et al.2014	14 / 14	24.5 ± 1.8	Acupuncture	Even reinforcing and reducing manipulation<br>the needle insertion depth is 2 - 3 cm.	3.0 T MEG	delta band
Fang et al.2012	10 / 11	22 – 28	Electroacupuncture	15 Hz, the needle insertion depth is 3 – 5 cm.	1.5T MRI	Bold signal
Fang et al.2006	11 / 3	23 ± 3	Acupuncture	Twirling Reinforcing and Reducing, the needle insertion depth is 2.2 cm.	1.5T MRI	Bold signal

He et al.2013	10	-	Electroacupuncture	-	3.0T MRI	Bold signal
Hu et al.2012	0 / 20	25.4	Acupuncture	Even reinforcing and reducing manipulation	1.5T MRI	Bold signal
Liu et al.2009	5 / 7	26.0 ± 1.1	Acupuncture	The needle insertion depth is 4 cm.	1.5T MRI	FC
Shang et al.2017	25 / 23	27.6 ± 4.3	Acupuncture	Reinforcing manipulation	1.5T MRI	Bold signal
Tan et al.2009	22 / 0	25 – 35	Acupuncture	Lifting-thrusting reinforcing and reducing	1.5T MRI	Bold signal
Tan et al.2013	6 / 6	23 – 27	Acupuncture	Twirling Reinforcing and Reducing, the needle insertion depth is 1- 1.5 cm.	3.0T MRI	Bold signal

Xiao et al.2008	9 / 7	24.8 ± 1.9	Acupuncture	Reinforcing manipulation, the needle insertion depth is 3 cm.	1.5T MRI	Bold signal
Yuan et al.2015	15 / 0	24 – 36	Acupuncture	Twirling Reinforcing and Reducing	MRI	Bold signal
Zhang et al.2016	7 / 11	20 – 30	Electroacupuncture	2Hz	1.5T MRI	Bold signal
Zhang et al.2011	22 / 0	26.0 ± 5.3	Acupuncture	Lifting-thrusting reinforcing and reducing	1.5T MRI	Bold signal

Abbreviations: ST36, the 36th acupoint of stomach meridian; ALFF, amplitude of low frequency fluctuations; fALFF, fractional amplitude of low frequency fluctuations; ReHo, regional homogeneity; FC, functional connectivity.

Table S2. Sample and imaging characteristics of the studies included in the resting-state activity analysis of GB34.

Study	Male/Female	Age (years)	Intervention modalities		Scanner	Measure
			Intervention	Operational details		
Huang et al.2010	6 / 6	25 ± 5	Electroacupuncture	4 Hz	1.5T MRI	Bold signal
Naa et al.2009	12 / 0	33.6 ± 6.2	Electroacupuncture	2Hz, Lifting-thrusting reinforcing and reducing, the needle insertion depth is 2 cm.	3.0T MRI	Bold signal
Yeo et al.2016	11 / 11	43.9 (25 - 66)	Acupuncture	Twirling Reinforcing and Reducing	3.0T MRI	Bold signal
Liu et al.2016	4 / 6	20 – 34	Acupuncture	Twirling Reinforcing and Reducing	3.0T MRI	ReHo

Fang et al.2006	11 / 3	23 ± 3	Acupuncture	Twirling Reinforcing and Reducing, the needle insertion depth is 2.2 cm.	1.5T MRI	Bold signal
Tian et al.2009	23 / 19	26.8 ± 5.0	Acupuncture	Twirling Reinforcing and Reducing, the needle insertion depth is 2.5 cm.	1.5T MRI	Bold signal

Abbreviations: GB34, the 34th acupoint of gallbladder; ReHo, regional homogeneity.

Table S3. Demographic information and Resting-state fMRI parameters of the discovery and validation datasets

Dataset characteristics	SLIM	FCP_ Cambridge_Buckner
**Sample size**	573	198
**Age (years)**	20.0 (19.9, 21.0)	19.0 (21.0, 22.0)
**Gender (F/M)**	316 / 256	75 / 123
**Scanner**	3.0T Siemens Trio	3.0T Siemens Trio
**Sequence**	GRE-EPI	GRE-EPI
**TR (ms)**	2000	3000
**Matrix size**	64 × 64	72 × 72
**Slices**	32	47
**Time points**	242	119

Age is expressed as median (P_{25}, P_{75}). Abbreviations: SLIM, Southwest University Longitudinal Imaging Multimodal Dataset; EPI, echo planar imaging; fMRI, functional magnetic resonance imaging; GRE, gradient echo; FCP, Functional Connectomes Project; TR, repetition time.

Sensitivity analysis

The sensitivity analysis of task-fMRI based study demonstrated high spatial consistency. For ST36, the Dice coefficient was 0.88 for the task-based subgroup. The activated network predominantly implicated the somatomotor network (SMN) (overlapping proportion: 35.58%), dorsal attention network (DAN) (16.32%), ventral attention network (VAN) (14.02%), visual (12.58%), default mode network (DMN) (8.25%), subcortical regions (8.18%), limbic (3.16%), frontoparietal control (1.97%) networks (Figure S1 A and C); For GB34, the Dice coefficient was 0.96 for the task-based subgroup. The activated network predominantly implicated the subcortical regions (overlapping proportion: 27.01%), VAN (26.84%), SMN (25.42%), DAN (9.26%), DMN (6.02%), frontoparietal control (2.61%), visual (2.13%), limbic (0.71%) networks (Figure S1 B and D);

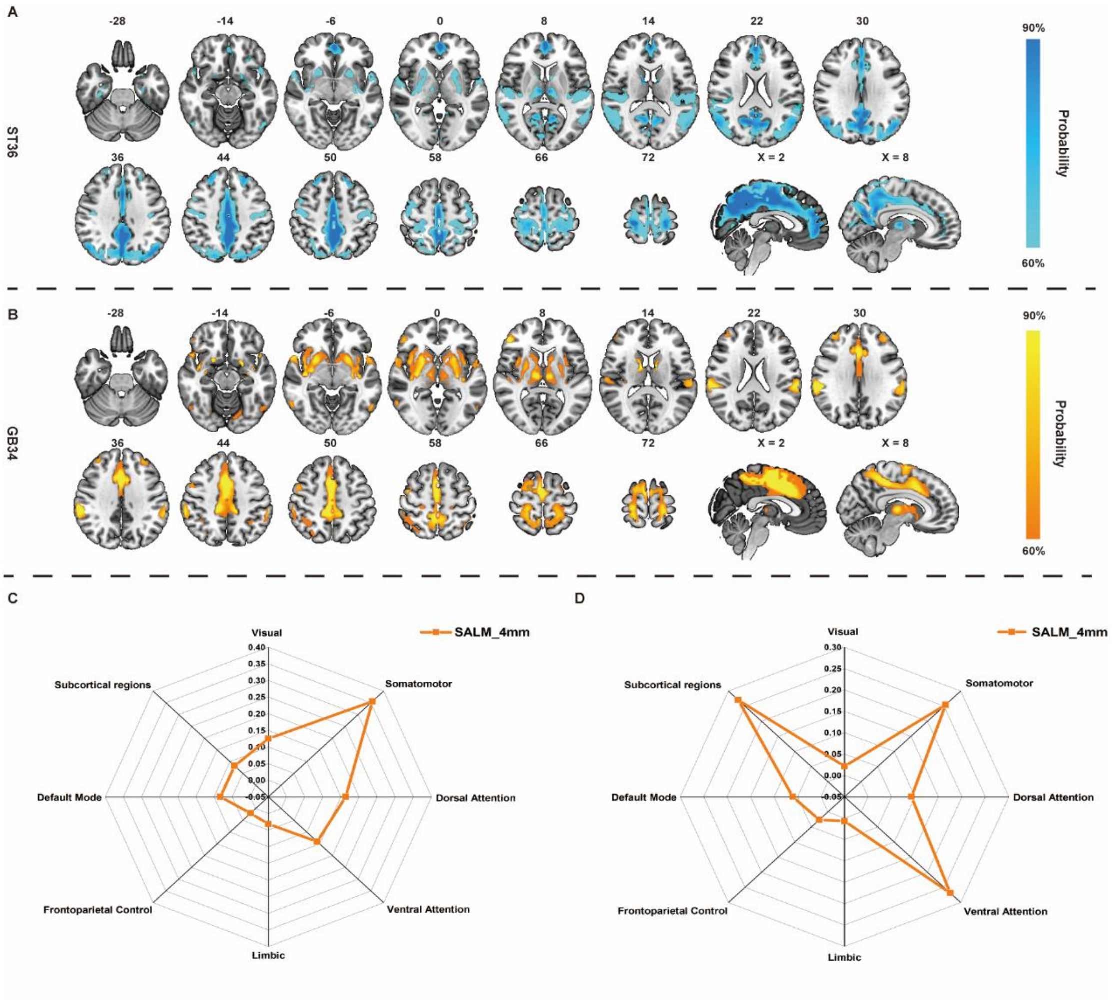

Figure S1. A and C. Brain activated network of ST36 in HCs on the task fMRI (Dice coefficient: 0.88); B and D. Brain activated network of GB34 in HCs on the task fMRI (Dice coefficient: 0.96).

Availability of data and materials

The data utilized in this study belong to the 1000 Functional Connectomes Project of Southwest University Longitudinal Imaging Multimodal (SLIM) dataset (https://fcon_1000.projects.nitrc.org/indi/retro/southwestuni_qiu_index.html) and Cambridge Buckner dataset (https://fcon_1000.projects.nitrc.org/fcpClassic/FcpTable.html). The analysis codes used in the article are publicly available at https://github.com/mfmaplestory/FCNM.
